# Impact of Annual versus Semiannual Mass Drug Administration with Ivermectin and Albendazole on Helminth Infections in Southeastern Liberia

**DOI:** 10.4269/ajtmh.21-0768

**Published:** 2021-11-22

**Authors:** Obiora A. Eneanya, Lincoln Gankpala, Charles W. Goss, Fatorma K. Bolay, Gary J. Weil, Peter U. Fischer

**Affiliations:** ^1^ Infectious Diseases Division, Department of Medicine, Washington University School of Medicine, St. Louis, Missouri;; ^2^ Division of Public Health and Medical Research, National Public Health Institute of Liberia, Charlesville, Republic of Liberia;; ^3^ Division of Biostatistics, Washington University School of Medicine, St. Louis, Missouri

## Abstract

We compared the impact of three rounds of annual and five rounds of semiannual mass drug administration (MDA) with albendazole plus ivermectin on helminthic infections in Liberia. Repeated annual cross-sectional community surveys were conducted between 2013 and 2019 in individuals of 5 years and older. Primary outcome was the change of infection prevalence estimates from baseline to month 36 (12 months after the last treatment). After three rounds of annual MDA, *Wuchereria bancrofti* circulating filarial antigen (CFA) and microfilaria (Mf) prevalence estimates decreased from 19.7% to 4.3% and from 8.6% to 0%, respectively; after semiannual MDA, CFA and Mf prevalences decreased from 37.8% to 16.8% and 17.9% to 1%, respectively. Mixed effects logistic regression models indicated that the odds of having Mf decreased by 97% (*P* < 0.001) at month 36 (similar odds for annual and semiannual MDA zones). A parallel analysis showed that the odds of CFA were reduced by 83% and 69% at 36 months in the annual and semiannual treatment zones, respectively (*P* < 0.001). *Onchocerca volvulus* Mf prevalence decreased slightly after multiple MDA rounds in both treatment zones. Reductions in hookworm and *Trichuris trichiura* prevalences and intensities were slightly greater in the annual treatment zone. *Ascaris lumbricoides* prevalence rates were relatively unchanged, although infection intensities decreased sharply throughout. Results show that annual and semiannual MDA were equally effective for reducing LF and soil-transmitted helminth infection parameters over a 3-year period, and reductions recorded at month 36 were sustained by routine annual MDA through month 72.

## INTRODUCTION

Neglected tropical diseases (NTDs) are a diverse set of 20 diseases that cause significant morbidity, primarily in the developing world.^1^ Several important NTDs are caused by helminth parasites that often share similar intervention and monitoring and evaluation strategies. Liberia is endemic for multiple helminthic NTDs.^2^ These include bancroftian filariasis (lymphatic filariasis [LF] caused by *Wuchereria bancrofti*), onchocerciasis, soil-transmitted helminth (STH) infections, and schistosomiasis.^3^^,^^4^

Community directed treatment with ivermectin (CDTi) targeting > 1 million people at risk for onchocerciasis commenced in Liberia in 2000 in endemic areas within all 15 endemic counties.^2^ Although these efforts recorded high geographical treatment coverage, transmission is still ongoing in all endemic foci. LF was mapped in 2010, and annual mass drug administration (MDA) for LF with ivermectin plus albendazole (IA) started in some areas in 2012. This often used the same CDTi infrastructure that had been used for ivermectin distribution.^2^^,^^5^ Schistosomiasis and STH control programs in Liberia provide praziquantel and albendazole to school-aged children. Unfortunately, many years of civil unrest (1990–2003) and the 2014 West Africa Ebola outbreak further weakened the fragile public health infrastructure in Liberia and interrupted MDA program activities.^1^^,^^6^ As MDA is still required to control and eliminate NTDs in Liberia, detailed information on the impact of MDA could help the country to plan future intervention activities.

There is no consensus regarding the relative value of annual versus semiannual MDA for helminthic NTDs. A simulation modeling study suggested that semiannual MDA could accelerate elimination of LF in West Africa,^7^^,^^8^ but field data were required to ground truth the model predictions. Therefore, we performed an integrated intervention and assessment project to test whether more frequent MDA could accelerate LF elimination in Liberia and to assess its impact on other co-endemic helminthic NTDs. Parasitological surveys were performed to assess the impact of MDA. The Liberian Ministry of Health assumed responsibility for MDA after our study was completed. A final parasitological survey was performed at 72 months to determine whether improvements observed in our study through 36 months were sustained over the next 3 years by routine annual MDA provided by the government.

## MATERIALS AND METHODS

### Study area.

**Figure 1.**
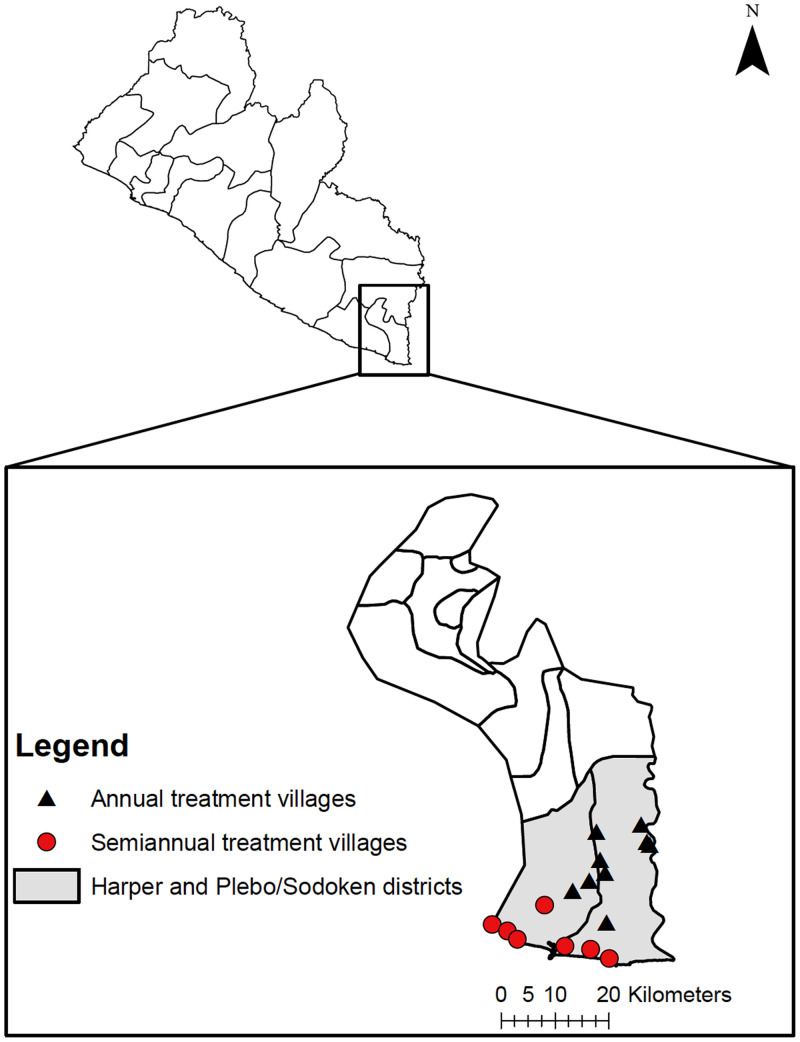
Maps of Liberia (top) and Maryland County (bottom). Study districts are shaded in gray and survey locations marked. This figure appears in color at www.ajtmh.org.

The study was conducted in 16 villages in Maryland County in southeastern Liberia (Figure 1). Harper and Plebo/Sodoken (combined population ∼61,000)^2^ are coastal districts within Maryland County that is bordered on the south by the Atlantic Ocean and on the east by Côte d’Ivoire. The weather in Liberia is warm and humid with a distinct dry and rainy season. The rainy season begins in May and ends in September, with annual rainfall average of 170 inches. Primary health centers provide basic medical care and maternity services. Anthelmintic drugs such as albendazole and mebendazole can be purchased without prescriptions in local pharmacies or markets.

### Mass drug administration.

Sixteen villages were divided into two treatment zones that received either annual or semiannual MDA. Treatment was delivered in Q3 2013, 2014, and 2015 in all study villages and additional rounds in Q1 2014 and 2016 (for villages that received semiannual MDA only). Seven villages closer to the coast received five rounds of semiannual MDA at 0, 6, 12, 18, and 24 months, whereas nine less accessible villages farther from the coast received annual MDA at 0, 12, and 24 months. The DOLF project (https://dolfproject.wustl.edu/) provided MDA through the 36-month time point. The Liberian Ministry of Health provided MDA in months 48 and 60 in all areas including other villages in the district that were not sites for this study. All eligible participants were offered directly-observed oral treatment with ivermectin (200 µg/kg, dosed using a dosing pole) plus albendazole (a fixed dose of 400 mg). Treatment compliance was estimated based on the proportion of the surveyed population that reported that they had swallowed the medications during the previous round of MDA. Our study area did not receive any previous MDA for LF or onchocerciasis, although a small proportion of the population had been treated with ivermectin at some point before the study. School-based schistosomiasis control using praziquantel was provided by the COUNTDOWN project between 2014 and 2018.^9^ Although NTD intervention was interrupted in many areas in Liberia by the Ebola outbreak in 2014, MDA was not affected in the Harper study villages.^10^

### Parasitological surveys.

Five annual cross-sectional parasitological surveys were conducted between 2013 and 2019. Community surveys were conducted at baseline and at yearly intervals (between May and June each year) through month 36 to assess the impact of MDA. We conducted a final follow-up survey at the 72-month time point (1 year after the last MDA provided by the Liberia Ministry of Health at month 60) to assess the sustainability of changes that occurred during our core study. Parasitological tests were performed in individuals ≥ 5 years of age without any evidence of acute illness or severe chronic disease were eligible to participate. Pregnant women were excluded. Individuals who were not eligible for MDA or who moved to the village within the last year were not included in the study.

#### Diagnostics for filarial infections.

Circulating filarial antigenemia (CFA) for LF was assessed in the first year of the study with the Binax Filariasis Now test (ICT, Alere, Scarborough, ME). CFA was detected with Filariasis Test Strips (FTS, Alere, Scarborough, ME) after the first year as the ICT test was being phased out.^11^ Both tests for CFA were independently read and scored by two readers. Scoring was based on a scale of 0–3 as previously described.^12^ If the results differed (uncommon), a final scoring decision was made by a supervisor.

Night blood smears were collected from persons with positive CFA test results to detect Mf by microscopy. A finger prick blood sample of 250 µL was collected into EDTA tubes between the hours of 8 pm and 11 pm. Blood smears were prepared the next morning by placing a total of 60 µL on glass slides in three horizontal lines. Slides were left to dry for 2 days, gently dehemoglobinized with distilled water for 3 minutes, air dried, fixed with methanol for 1 minute, and stained with Giemsa for 15 minutes. Trained technicians examined the stained slides for the presence of Mf by microscopy.

Because the study area was considered to be non-endemic for onchocerciasis based on Ministry of Health mapping, the study area was ivermectin-naïve, and skin snips surveys were not planned for our study. However, clinical onchocerciasis cases were detected in the communities during the baseline survey. For subsequent surveys, we expanded the study to include collection of skin snips for detection of *Onchocerca volvulus* Mf. Therefore, onchocerciasis diagnosis was first included in villages receiving annual MDA in the first follow-up survey (month 12). After a relatively high prevalence of *O. volvulus* skin Mf was detected in this area, we decided to extend onchocerciasis diagnosis for the entire study area from the second follow-up (month 24) survey onwards. *Onchocerca volvulus* infections were detected by skin snip testing. Briefly, one skin snip was taken from each posterior iliac crest using a sterile 2 mm Holth-type corneoscleral punch (Everhards GmbH, Meckenheim, Germany). Snips were incubated in 100 µL phosphate-buffered saline for 24 hours in flat-bottomed 96-well microtiter plates at room temperature. Emerging microfilariae were detected and counted by bright-field microscopy (×40).

#### Detection of STH and intestinal schistosomiasis infections.

Study participants were asked to produce stool samples on a clean sheet of paper and transfer a walnut-sized piece into the container. Samples were labeled with a barcode and the name of the participant. Samples were kept cool and transported to the field laboratory where they were tested by duplicate Kato-Katz smears for detection of helminth ova.^13^ Slides were read within 60 minutes after preparation by two microscopists. For quality control, a random selection of 10% of the slides were reexamined by a senior microscopist, and results were matched with the prior readings. In rare instances of a major discrepancy between the duplicate readings, all slides from that day’s stool collection were reexamined.

### Data management and statistical analysis.

Demographic data were directly entered into the Epi Info for Mobile Devices App (Centers for Disease Control and Prevention, Atlanta, GA). Deidentified, encrypted data were transferred to an Azure cloud server (Microsoft, Seattle, WA). Laboratory results were recorded on paper forms with barcode identifiers and subsequently transferred with double data entry into Epi Info.^14^ Data were downloaded from the cloud server, converted into Microsoft Excel, and cleaned before analysis.

Sample size calculations were made using binomial power calculator (using alpha = 0.05, two-tailed tests, and power = 0.80). These sample sizes were chosen to provide confidence that measured prevalence rates will be below specified limits based on assumptions regarding true prevalence rates. The sample size number per sentinel site (village) is close to the overall minimum target sample size of 335 needed to show that filarial antigen rates are less than 2% (with 95% confidence and 80% power, assuming an expected rate of 0.5%, which is an LF elimination target). The study aimed to enroll at least 2,500 participants. The sample size analysis considered various contingencies to assess robustness of the sample size estimates.

Participant’s exact age at time of survey was recorded. However, for the purpose of analysis, ages were grouped into six categories: ≤ 10 years, 11–20 years, 21–30 years, 31–40 years, 41–50 years, and > 50 years. Eggs per gram (epg) was used as a measure of STH infection intensity, and these values were classified as low, moderate, or high intensities based on WHO thresholds.^15^ Onchocerciasis skin snip intensity classes were defined as < 10 Mf/mg skin for light infection, 11–30 Mf/mg skin for moderate infection, and > 30 Mf/mg skin for heavy infection.

The primary outcome (differences in % infection prevalence from baseline to the 36-month time point) was analyzed by comparison of cross-sectional data. Data from month 72 were compared with results from month 36 to detect changes that may have occurred after the primary study endpoint. Cross-sectional surveys for every time point were considered to be independent, so unpaired statistical tests were used. Unadjusted prevalence estimates were calculated (with 95% exact CIs) for each treatment zone and survey. Changes in infection prevalence between MDA rounds were assessed by Fisher’s exact test. Mixed effects logistic regression models were also used to evaluate differences in the odds of infection between the treatment zones and changes in the odds of infection over time. Village was treated as a random effect to account for potential correlation among subjects within a locality. We assessed the treatment regimen, time, and their interaction. The interaction term was excluded from the final model if it was not significant (*P* < 0.05), otherwise it was retained. Both age and sex were included as covariates in all of the mixed-effects models.

The geometric mean of infection intensity (Mf count or epg) was calculated with data from persons with positive infections, whereas all persons tested were considered for calculating arithmetic mean. The community microfilarial load (CMFL) was calculated as the geometric mean number of Mf/mL of blood (for LF) and Mf/mg skin snip (for onchocerciasis) using a log(*X*_1_+1) transformation, where *X* is the microfilaremia count of all subjects in the study including those without Mf.^16^^–^^18^ The CMFL analysis for onchocerciasis was restricted to subjects aged 19 and older. A univariable logistic regression model was used to identify risk factors for filarial infection (baseline data only). Results are presented as unadjusted odds ratios (aOR) with 95% confidence limits. *P* values < 0.05 were considered significant, and statistical analyses were conducted using the statistical software package R (v 4.0.3)^19^ and SAS (v 9.4, SAS Inst. Inc., Cary, NC).

### Ethical approval.

The study protocol was reviewed and approved by institutional review boards at Washington University School of Medicine (Institutional Review Board ID number: 201107185) and at the University of Liberia (FWA00004982). Researchers met with district officials and village leaders prior to the field study. Oral informed consent was obtained for each participant. Enrollment of minors required their consent plus informed consent from at least one parent or guardian. All participant data were de-identified before they were uploaded into the cloud server.

## RESULTS

### Description of study population.

A total of 7,838 participant contact events was recorded during the study period, of which 4,271 were in the treatment zone that received annual MDA (Table 1). The average reported treatment compliance and use of insecticide treated bed nets was < 45% and < 35% respectively during the years of the trial. Latrine ownership can be used as a proxy for socioeconomic status, and was higher in the zone that received annual MDA compared with the coastal zone that received semiannual MDA.

**Table 1:** Characteristics of persons enrolled during the study period

Variable	Annual treatment zone	Semiannual treatment zone
Total number of participant contact events	4,271 (54.5%)	3,567 (45.5%)
Gender (male)	2,413 (56.5%)	1,734 (48.6%)
Median age, years (range)	21 (5–89 years)	22 (5–94 years)
Treatment compliance*	43% (range 27–66%)	39.2% (range 35–49%)
Baseline (month 0)	NA	NA
Follow-up 1 (month 12)	38.1%	35.1%
Follow-up 2 (month 24)	66.4%	49.1%
Follow-up 3 (month 36)	44.1%	37.3%
Follow-up 4 (month 72)	27.1%	35.0%
Bed net usage†	1,324 (31%)	1,230 (34.5%)
Door/window screen in houses	427 (10%)	228 (6.4%)
Latrine ownership	3,588 (84%)	1,698 (47.6%)

*Average recall 6–12 months after treatment. Treatment compliance was defined as individuals who reported swallowing drugs.

†Bed net usage was defined as persons who slept under bed net the night prior to survey.

### Factors associated with *W. bancrofti* infection.

**Figure 2.**
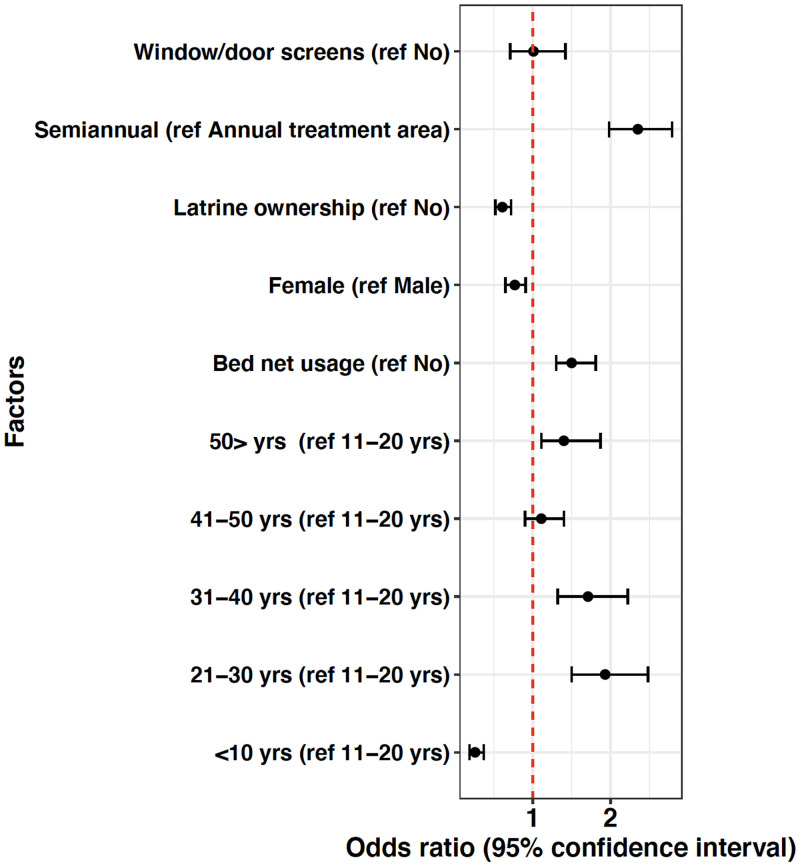
Univariable analysis of risk factors for filariasis (based on baseline data). The dashed red line indicates an odds ratio of 1. This figure appears in color at www.ajtmh.org.

A univariable analysis was used to identify risk factors of filarial infection at baseline (measured as positive CFA tests). Figure 2 shows that window/door screens on houses had no effect on whether persons tested positive for filariasis. Prior to any MDA, persons who lived in the villages that later received semiannual MDA had higher odds of infection (odds ratio: 2.35, 95% CI 1.98, 2.79). Persons who had latrines in their houses had lower odds of infection (odds ratio: 0.61, 95% CI 0.52, 0.72). Females also had lower odds of infection compared with males (odds ratio: 0.77, 95% CI 0.65, 0.91). Children aged 10 years and below had lower odds of infection (compared with the reference age group 11–20 years), whereas all older age groups had higher odds when compared with the reference group. Window/door screens were not statistically significant (*P* = 0.94). All other risk factors were significant with *P* < 0.05.

### Impact of annual versus semiannual MDA on filarial infections.

Table 2 shows that the baseline *W. bancrofti* infection prevalence estimates (CFA and Mf) were higher in the semiannual treatment zone. After three rounds of MDA, CFA and Mf prevalence estimates in the annual MDA treatment zone decreased by 78% (from 19.7% to 4.3%) (*P* = 0.023) and by 100% (from 8.6% to 0%) (*P* = 0.007), respectively. CFA and Mf prevalence estimates in the semiannual MDA treatment zone decreased by 55% (from 37.8% to 16.8%) (*P* = 0.028) and by 94% (from 17.9% to 1%) (*P* < 0.001), respectively, after five rounds of MDA. Thus, both treatment schedules reduced CFA and Mf prevalence estimates dramatically over a period of 36 months. Annual MDA was more effective for reducing geometric mean Mf/mL and CMFL (*P* < 0.001 and 0.048, respectively) when compared with semiannual MDA (*P* < 0.001 and 0.438, respectively), but very few persons were Mf positive in the 36 months (follow-up 4) post-MDA survey.

**Table 2:** Impact of mass drug administration on the prevalence of lymphatic filariasis

Treatment zone	Timing of the survey	Number of participants (*N*)	CFA prevalence (95% CI)	Mf prevalence (95% CI)	Geometric mean Mf/mL	CMFL
Annual MDA	Baseline (month 0)*	1,125	19.7 (17.0, 22.6)	8.6 (6.8, 10.8)	100.6 (69.6, 145.3)	4.4 (4.1, 4.7)
	Follow-up 1 (month 12)	798	13.6 (11.2, 16.3)	2.5 (1.3, 4.4)	167.8 (72.9, 386.2)	4.9 (4.1, 5.8)
	Follow-up 2 (month 24)	759	15.1 (12.0, 18.7)	2.3 (1.2, 3.8)	205.4 (51.2, 823.8)	5.0 (3.8, 6.4)
	Follow-up 3 (month 36)	650	4.3 (2.6, 6.6)	0 (0, 0.6)	0 (0, 0.4)	0 (0, 0.3)
	Follow-up 4 (month 72)†	939	3.8 (2.6, 5.3)	0.1 (0, 0.6)	1 Mf+ person	0.016 (0, 0.073)
Semiannual MDA	Baseline (month 0)*	1,000	37.8 (34.2, 41.6)	17.9 (15.1, 21.0)	61.3 (48.9, 76.8)	4.0 (3.8, 4.2)
	Follow-up 1 (month 12)	635	23.1 (19.9, 26.6)	4.4 (2.8, 6.4)	159.1 (88.8, 285.2)	4.9 (4.3, 5.5)
	Follow-up 2 (month 24)	594	24.6 (21.1, 28.5)	2.6 (1.3, 4.6)	173.3 (88.1, 340.8)	5.1(4.4, 5.8)
	Follow-up 3 (month 36)	677	16.8 (13.5, 20.4)	1.0 (0.3, 2.4)	97.9 (28.7, 334.0)	3.4 (3.3, 4.7)
	Follow-up 4 (month 72)†	661	7.5 (5.5, 9.9)	0.5 (0.09, 1.3)	3 Mf+ persons	0.5 (0.38, 0.72)

CFA = circulating filarial antigenemia; CMFL = community microfilarial load; FTS = filariasis test strips; ICT = immunochromatographic test; MDA = mass drug administration; Mf = microfilaria.

*CFA was detected by ICT at baseline. FTS was used for all follow-up surveys.

†Both treatment zones received once/year MDA from the Liberia Ministry of Health from months 36 to 60.

Mixed effects logistic regression models were used to assess differences in odds of Mf and CFA infection between treatment groups over time (Table 3). The treatment-by-time interaction was not significant in the model for Mf infection (*P =* 0.70) and was excluded from the final Mf model. Our results indicated that the odds of infection (measuring for Mf) in the semiannual treatment group was 2.75 higher than the annual treatment group (*P* = 0.058). Additionally, compared with baseline there was a strong overall decline in the odds of Mf infection over time of 82% by month 12, 87% by month 24, and 97% by month 36 (*P* < 0.001; Table 3). Similarly, results from the CFA model showed a strong decline in CFA infection over the course of the study, however, in contrast to the Mf model there was more variability in treatment differences over time as indicated by the significant treatment-by-time interaction (*P* = 0.048). Relative to baseline CFA infection, the decline in the odds of CFA positivity ranged between 56% and 83% for the annual treatment zone, and 47%–69% for the semiannual treatment zone (Table 3). When we compared CFA positivity between the treatment zones at each time point, we found that the odds of infection was 2–3 times higher in the semiannual treatment zone compared with the annual treatment zone in the baseline (aOR, 2.19; 95% CI, 0.99–4.85; *P* = 0.054), 12 months (aOR, 2.61; 95% CI, 1.15–5.92; *P* = 0.024), and 24 months (aOR 2.12; 95% CI, 0.94–4.8; *P* = 0.069), and increased to an odds ratio of 4 at 36 months (aOR, 4.02; 95% CI, 1.71–9.46; *P* = 0.003).

**Table 3:** Adjusted odds ratios and 95% CIs from mixed effects logistic regression models comparing 12, 24, and 36 months to baseline for Mf and CFA infection

Outcome	Treatment zone	Comparison	Adjusted odds ratio (95% CI)	*P value*
Mf	NA	12 months vs. Baseline	0.18 (0.13, 0.27)	< 0.001
		24 months vs. Baseline	0.13 (0.08, 0.19)	< 0.001
		36 months vs. Baseline	0.03 (0.01, 0.06)	< 0.001
CFA	Annual	12 months vs. Baseline	0.44 (0.33, 0.59)	< 0.001
		24 months vs. Baseline	0.54 (0.41, 0.7)	< 0.001
		36 months vs. Baseline	0.17 (0.12, 0.25)	< 0.001
	Semiannual	12 months vs. Baseline	0.53 (0.41, 0.68)	< 0.001
		24 months vs. Baseline	0.52 (0.41, 0.66)	< 0.001
		36 months vs. Baseline	0.31 (0.24, 0.4)	< 0.001

Mf = microfilaria; CFA = circulating filarial antigenemia.

The nonsignificant treatment-by-time interaction was removed from the Mf model whereas there was a significant treatment-by-time interaction for the CFA model. Accordingly, odds ratio estimates are aggregated across treatments for Mf and by treatment zone for CFA.

**Figure 3.**
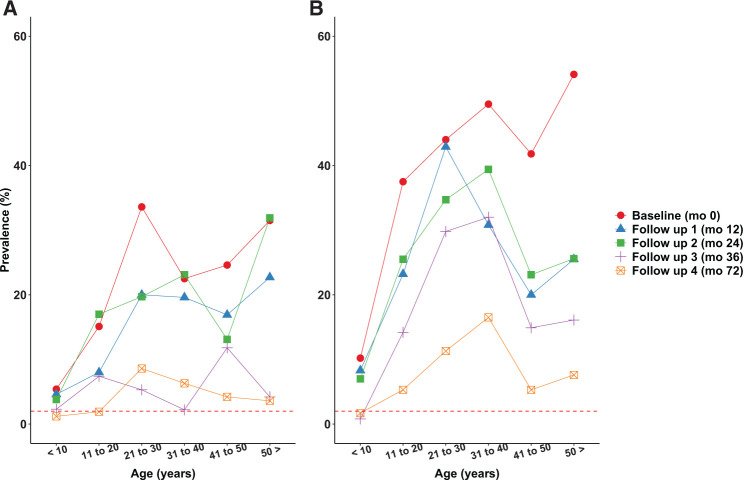
(**A** and **B**) Age-prevalence profiles for circulating filarial antigenemia (CFA) by treatment zone before and after mass drug administration. The dotted red line indicates the 2% pre-transmission assessment surveys (TAS) prevalence target. This figure appears in color at www.ajtmh.org.

**Figure 4.**
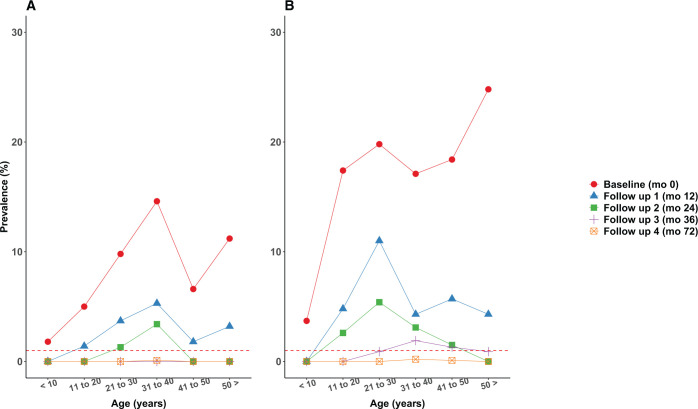
(**A** and **B**) Age-prevalence profiles for microfilaremia (Mf) by treatment zone before and after mass drug administration. The dotted red line indicates the 1% pre-transmission assessment surveys (TAS) prevalence target. This figure appears in color at www.ajtmh.org.

Figures 3 and 4 show age-prevalence profiles for *W. bancrofti* infection before and after MDA. Although the baseline prevalence estimates for both infection markers were higher in the semiannual MDA treatment zone, the patterns are similar in the two treatment zones with peak prevalence estimates in young adults. The shapes of the patterns persisted over time as prevalence estimates decreased after MDA. CFA prevalences remained above the 2% pre-transmission assessment surveys (TAS) target in most age groups after MDA at 36 and 72 months in both treatment zones. In contrast, most age groups had Mf prevalence estimates below the pre-TAS target of 1% at 36 and 72 months in both treatment zones. There was no evidence that LF infection prevalence increased in the 2 years after the national NTD program assumed responsibility for MDA in the study area.

Table 4 shows the effect of MDA on *O. volvulus* Mf prevalence and infection intensity by treatment zone. MDA decreased skin Mf prevalence estimates at 36 months in both treatment zones, but prevalence estimates rebounded between 36 and 72 months, although these were not statistically significant (*P* = 0.296 and 0.178 in the annual and semiannual treatment zones, respectively). *Onchocerca volvulus* Mf prevalences were 26.6% and 17.0% in the annual and semiannual MDA zones after 6 or 8 rounds of MDA, respectively. The geometric mean Mf/mg skin and CMFL remained stabled throughout in the annual treatment zone, whereas these increased in the semiannual treatment zone. Overall, onchocerciasis infection intensities were mostly light infections (i.e., < 10 Mf/mg skin).

**Table 4:** *Onchocerca volvulus* microfiladermia prevalence estimates and infection intensities after MDA by treatment zone. No assessment for onchocerciasis was done at baseline in both treatment zones and at the first follow-up in the semiannual MDA zone

	Follow-up 1 (month 12)	Follow-up 2 (month 24)	Follow-up 3 (month 36)	Follow-up 4 (month 72)	*P* value (month 12–72)
Annual MDA (*N* = 2517)	*N* = 729	*N* = 473	*N* = 444	*N* = 871	
Prevalence (95% CI)	31.8 (27.9, 35.8)	25.6 (20.0, 31.7)	19.5 (15.7, 23.9)	26.6 (22.9, 30.6)	0.399
Geometric Mf/mg skin (95% CI)	9.4 (7.7, 11.3)	3.2 (2.4, 4.6)	9.0 (6.4, 12.7)	11.2 (8.1, 13.3)	0.221
Community Mf load (CMFL)	2.0 (1.9, 2.2)	1.3 (1.1, 1.6)	2.0 (1.8, 2.4)	2.1 (1.7, 2.6)	0.974
Class of intensity (%)					
Light (< 10 Mf/mg skin)	50 (44.8, 53.8)	84.5 (77.9, 90.1)	59.2 (45.9, 63.8)	71 (67.3, 83.1)	0.018
Moderate (11–30 Mf/mg skin)	31 (25.9, 35.7)	10.3 (6.7, 13.2)	26.8 (20.9, 32.1)	11.9 (9.8, 13.4)	< 0.001
Heavy (> 30 Mf/mg skin)	19 (15.1, 25.9)	5.2 (3.2, 7.0)	14.1 (12.9, 17.5)	17.1 (14.9, 21.8)	0.044
					***P value *(month 24–72)**
Semiannual MDA (*N* = 1,523)		*N* = 425	*N* = 485	*N* = 613	
Prevalence (95% CI)	–	29.6 (24.0, 35.8)	10.0 (7.1, 13.4)	17.0 (12.6, 20.8)	0.005
Geometric Mf/mg skin (95% CI)	–	5.1 (3.7, 6.9)	11.8 (6.8, 20.1)	12.4 (9.4, 18.2)	0.186
Community Mf load (CMFL)	–	1.6 (1.4, 1.9)	2.3 (1.9, 3.0)	2.5 (1.7, 2.8)	0.901
Class of intensity (%)	–				
Light (< 10 Mf/mg skin)	–	66.7 (53.4, 76.4)	54.3 (43.4, 59.1)	82.4 (77.3, 91.0)	0.054
Moderate (11–30 Mf/mg skin)	–	27.8 (23.9, 34.1)	17.1 (15.9, 20.2)	13.2 (10.9, 16.3)	0.052
Heavy (> 30 Mf/mg skin)	–	5.6 (4.2, 7.0)	28.6 (22.3, 34.3)	4.4 (2.9, 6.7)	< 0.001

CMFL = circulating filarial antigenemia; MDA = mass drug administration; Mf = microfilaria.

### Impact of annual versus semiannual MDA on STH infections.

**Figure 5.**
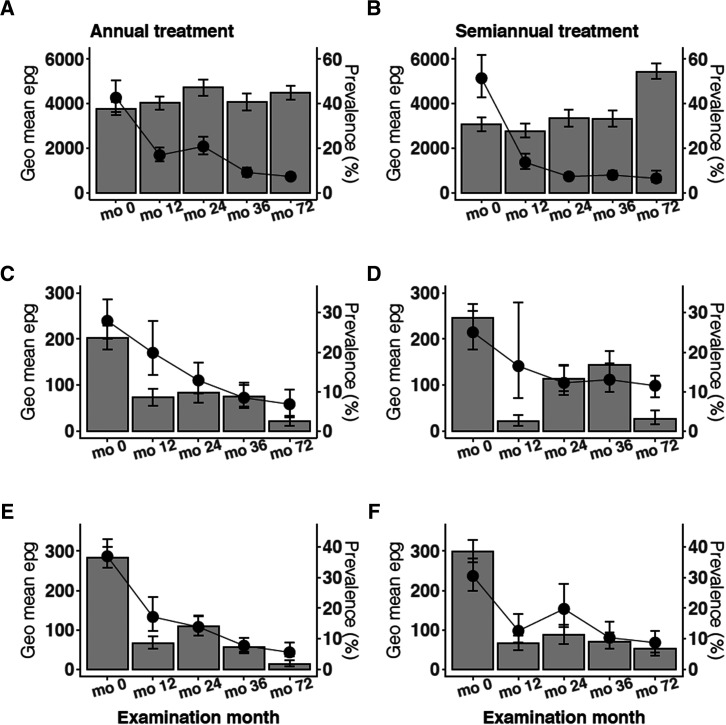
Impact of annual vs. semiannual mass drug administration on helminth infections. (**A** and **B**) *Ascaris lumbricoides* infection in annual and semiannual treatment zones, respectively. (**C** and **D**) Hookworm infection in annual and semiannual treatment zones, respectively. (**E** and **F**) *Trichuris trichiura* infection in annual and semiannual treatment zones, respectively. Prevalence is represented in bar graphs. Geometric mean eggs per gram are shown in line graphs. Month (mo); Geometric mean eggs per gram (Geo mean epg).

STH survey results are summarized in Figure 5. *Ascaris,* hookworm, and *Trichuri*s infections were present with similar prevalence estimates in both treatment zones at baseline. Annual and semiannual MDA had similar effects in both treatment zones. MDA had little effect on *Ascaris* prevalence (*P* = 0.349 and 0.323 for annual and semiannual treatment zones respectively), but it was associated with decreases in infection intensities (geometric mean epg) (*P* < 0.001 in both annual and semiannual treatment zones). In contrast, MDA decreased prevalences and geometric mean epg for hookworm (*P* < 0.001) and *Trichuris* (*P* < 0.001) in both treatment zones. Supplemental Table 1 provides information on the number of participants tested for STH in each treatment zone per survey year, prevalence estimates, and infection intensities (arithmetic mean and geometric mean egg counts). Almost all STH infections detected in the final survey at month 72 were of low intensity (Supplemental Table 2).

### *Schistosoma mansoni* infection in the study area.

Baseline prevalence estimates for *S. mansoni* were < 1% in both treatment zones (Table 5). However, prevalence estimates increased slowly by 36 months in the inland annual treatment zone. Schistosomiasis prevalence increased dramatically to more than 45% in both treatment zones between months 36 and 72. Geometric mean epg levels remained relatively unchanged in both treatment zones (*P* < 0.001 in annual and semiannual treatment zones). Schistosomiasis treatment was not included as part of this intervention study.

**Table 5:** Changes in *Schistosoma mansoni* prevalence over time by treatment zone

Treatment zone	Treatment round	Number of participants	Prevalence of *S*. *mansoni* (95% CI)	*P* value (compared with baseline)	Geometric mean *epg *(95 CI)
Annual MDA	Baseline (month 0)	792	0.8 (0.3, 1.7)		120.6 (51.7, 281.1)
	Follow-up 1 (month 12)	729	5.6 (4.1, 7.6)	0.058	69.1 (53.3, 89.5)
	Follow-up 2 (month 24)	473	8.0 (5.7, 10.9)	0.015	88.1 (61.9, 125.8)
	Follow-up 3 (month 36)	444	9.9 (7.3, 13.1)	0.005	49.3 (37.7, 64.5)
	Follow-up 4 (month 72)	702	48.2 (45.0, 52.5)	< 0.001	80.1 (64.3, 93.4)
Semiannual MDA	Baseline (month 0)	698	0.1 (0.001, 0.9)		36.6 (23.2, 42.5)
	Follow-up 1 (month 12)	624	0.2 (0.001, 0.9)	0.851	207.4 (168.3, 221.4)
	Follow-up 2 (month 24)	425	0.5 (0.05, 1.7)	0.606	98.4 (78.8, 118.3)
	Follow-up 3 (month 36)	485	3.3 (1.9, 5.3)	0.830	59.3 (37.7, 93.1)
	Follow-up 4 (month 72)	467	47.6 (43.8, 53.0)	< 0.001	74.5 (63.2, 88.4)

MDA = mass drug administration.

## DISCUSSION

This study has provided a comprehensive assessment of the impact of three annual rounds and five semiannual rounds of MDA with IA on major helminthic NTDs in Liberia. In addition, results from the follow-up survey 72 months after the first round of MDA allowed us to determine whether improvements observed at 36 months were sustained after two additional rounds of annual MDA provided by the Liberia Ministry of Health.

Our study showed that annual MDA was as effective as semiannual MDA for reducing LF infection markers over a period of 36 months. These results with bancroftian filariasis are similar to those reported from a similar study of the effects of annual and semiannual MDA on brugian filarias in Indonesia.^20^ Preliminary results from these studies (in Liberia and Indonesia)^20^ and other similar studies not yet published were considered by the LF Guidelines Development Group for recommending annual MDA for LF elimination.^21^ Annual MDA using IA is recommended for LF control except for areas co-endemic with loiasis, where semiannual albendazole MDA is recommended.^21^

Although the odds of CFA at month 36 was higher in semiannual MDA zone than in the annual MDA zone, this may have been as a result of the higher baseline infection CFA prevalence in that zone and the fact that CFA often remains detectable in the blood of infected persons after multiple rounds of treatment with IA. Furthermore, the semiannual MDA zones had higher CFA test scores from baseline through the duration of the study (Kruskal-Wallis test; *P* < 0.001), and studies have shown that higher test scores take longer to clear.^22^

Sparse data were available on LF prevalence in southeastern Liberia prior to our study. A survey documented an Mf prevalence of 37% in coastal Maryland County (which includes Harper district) circa 1972.^23^ Some 40 years later, our survey documented a baseline Mf prevalence of 18% in this area. This study also showed that Mf prevalence decreased much faster than CFA prevalence after MDA with IA. Both treatment zones satisfied the WHO pre-TAS target for Mf (< 1%) but not for CFA (< 2%) at 72 months. These results and results from other studies show that WHO’s pre-TAS CFA target is more stringent than their pre-TAS Mf target^24^; use of the more conservative CFA target can be expected to require additional rounds of MDA beyond those required to reduce Mf prevalence below 1%.

It is interesting that the strong declines in LF prevalence in our study area were achieved with relatively low MDA compliance rates in both treatment zones. Many studies have shown that high treatment compliance is a key factor for successful MDA programs.^25^^,^^26^ It is possible that inaccurate recall led to underestimated compliance rates in this study, because compliance data were collected during surveys that were conducted 6–12 months after each round of MDA. Also, population figures for our study area may have been inflated by inclusion of persons who are considered village/family members who no longer reside there. However, data from the Liberia Ministry of Health NTD report for Maryland County in 2019 show therapeutic and geographical coverage rates of 86% and 97%, respectively.^27^ Taken together, compliance rates obtained in our surveys may have been inaccurately low. We suggest that future studies should perform a study-specific census of actual residents (for better estimates of denominators) as well as separate compliance surveys 2 or 3 months after MDA. These will reduce the chances of recall bias and provide more accurate adherence data. It is also interesting that more frequent MDA did not result in improved reported cumulative MDA compliance or more rapid reduction in LF infection parameters. Taken together, these results suggest that annual MDA with IA can be quite effective for LF elimination. If programs can manage adequate coverage with annual MDA, there is no need to spend additional resources to deliver a second round per year.

Adult males had higher LF prevalence estimates than adult females at baseline, and this difference persisted after MDA despite similar MDA compliance in males and females (data not shown). The higher baseline prevalence in adult males may be as a result of their higher exposure to bites from infective mosquitoes. Equivalent MDA compliance by gender may not be sufficient to overcome the increased exposure to infection that men experience. Regardless of the cause, it is important for MDA programs to ensure high coverage of adult males who account for a disproportionate burden of filarial infection in many endemic areas.^28^

We found that LF infections were more common in adults than in children. This is probably because adults have had more time to become infected. Higher prevalence in young adults compared with older people is more difficult to explain. It may reflect age related differences in exposure to mosquitoes or gradual development of partial immunity to infection. Individuals’ reported use of bed nets was not linked to infection prevalence. This is in contrast to results reported from a study in the Republic of the Congo, where bed net usage appeared to be protective, as has been previously reported elsewhere.^29^ Our result is also contrary to the notion that bed nets plus MDA should accelerate the interruption of LF transmission in Africa^30^ and Papua New Guinea.^31^ However, bed net usage data in this study may have been inaccurate, because it was entirely based on whether participants reporting having used a bed net during the night before the survey. It is interesting that access to a private household latrine was associated with a reduced risk of LF infection in this study. Tusting et al.^32^ demonstrated that ownership of a private latrine within the household might serve as a proxy for improved housing, improved sanitation and hygiene, and higher socioeconomic status, all of which have been associated with reduced risk of LF infection.^33^ This may also explain the negative association between latrine access and LF in our study.

Our study area was previously considered to be non-endemic for onchocerciasis.^2^ However, we observed persons with nodules and clinical disease during the baseline survey and decided to investigate this further in subsequent surveys. Our study documented mesoendemic (i.e., skin Mf prevalence between 30% and 60%) onchocerciasis, and baseline skin Mf prevalence in the study area was probably at least 30%. This is not surprising, because a recent geospatial modeling study for onchocerciasis in Côte d’Ivoire also showed high infection rates in areas bordering our study sites in Liberia.^34^ The problem of incomplete or inaccurate onchocerciasis mapping data is not limited to Liberia. Onchocerciasis elimination mapping now underway in Africa is certainly needed.^35^

Persistence of onchocerciasis during the 72-month survey was expected; although ivermectin is an effective microfilaricide, its effects on adult *O. volvulus* worms are only temporary.^36^^,^^37^ In this study, 29% and 18% of surveyed participants had *O. volvulus* Mf densities of more than 10 Mf per mg of skin 12 months after six or eight rounds of MDA with IA. Thus, onchocerciasis is much more difficult to eliminate than LF by MDA with ivermectin (alone or combined with albendazole). Kamgno et al. in their study in Cameroon observed persistent mesoendemic onchocerciasis despite more than 15 years of community-directed treatment with ivermectin.^38^

STHs are endemic in all counties of Liberia, but recent detailed data are limited.^2^ Hookworm was reported to be the most common species in the past; more than 90% of school children were infected with hookworm in coastal Maryland County in 1972.^39^ Our study found a lower prevalence of hookworm (even prior to MDA). This may reflect improvements in sanitation and hygiene and/or distribution of anthelmintics to school aged children. Infection intensities for all three STH infections decreased after MDA. Annual and semiannual MDA had similar beneficial effects on hookworm and *Trichuris* prevalence in our study. In contrast, MDA had little effect on *Ascaris* prevalence. STH prevalence and intensity behaved differently after MDA. The use of prevalence as a surrogate for intensity may be misleading especially in areas that have received MDA.^40^ Furthermore, prior studies have shown that reinfections are common after MDA^41^ and this may happen more quickly for *Ascaris* than for hookworm and *Trichuris.* It is interesting that improvements in STH parameters that were observed at 36 months were still present at 72 months. This suggests that the Liberia Ministry of Health achieved good compliance for their annual MDA with albendazole and ivermectin between months 36 and 60.

MDA can be an effective STH control intervention, but MDA alone will not eliminate STH infections in most endemic areas. For effective control of STH infections, the WHO recommends an integrated approach, which includes improved sanitation, hygiene education, and preventive chemotherapy, with good coverage. Improved sanitation is particularly vital for long-term suppression of endemicity levels. Although we did not implement sanitation, hygiene education, or behavior change interventions, our prolonged presence in these communities as well as extensive sensitization activities may have played an important role in educating the community on the importance of improved hygiene on infection prevention measures.

Intestinal schistosomiasis is highly prevalent in many areas of Liberia. Although MDA with praziquantel was not provided by our project, Liberia was part of the COUNTDOWN project that provided school-based schistosomiasis MDA from 2014 to 2018.^9^ At baseline, the community prevalence estimates for intestinal schistosomiasis in our study areas were less than 1%, and they were still less than 10% in both areas at month 36. The very high prevalence estimates of almost 50% in both study areas at month 72 (in 2019) were an unpleasant surprise. Cessation of the COUNTDOWN project may have played a role. Strategies recommended by Kayuni et al.^42^ for investigating community outbreaks of intestinal schistosomiasis might be useful for identifying the factors responsible for the rapid emergence of *S. mansoni* in this area.

In conclusion, this study documented dramatic changes in helminth infection prevalence estimates over time following introduction of MDA with IA. However, the impact of MDA varied for different helminth species. Annual MDA was just as effective as semiannual MDA for LF, and our results suggest that annual MDA should be sufficient to achieve LF elimination in areas of West Africa with characteristics that are similar to those in our study area. We also demonstrated that annual and semiannual MDA for LF had significant and similar beneficial effects on STH infection prevalence and intensity that were sustained after responsibility for MDA was handed off to the Liberia Ministry of Health. However, MDA will not be sufficient to eliminate STH. Finally, our study demonstrated that annual and semiannual MDA with IA had limited long-term effects on onchocerciasis prevalence over the time frame of this study. More years of MDA or better treatments will be required to eliminate onchocerciasis.

## Supplemental Material


Supplemental materials
