# Supplementary material for: Impact of Annual versus Semiannual Mass Drug Administration with Ivermectin and Albendazole on Helminth Infections in Southeastern Liberia
Source: Am J Trop Med Hyg. 2021 Nov 22;106(2):700–9. doi: 10.4269/ajtmh.21-0768 (PMC8832944; doi:10.4269/ajtmh.21-0768)
Supplement: Supplementary file 1 [file tpmd210768.SD1.pdf]

Supplementary 1: Impact of treatment on the prevalence and intensity of soil-transmitted helminth infections

Treatment zone	Treatment Round	Number of subjects (*N*)	Prevalence of *A. lumbricoides* (95% CI)	Arithmetic mean *epg* of *A. lumbricoides* (95% CI)	Geometric mean *epg* of *A. lumbricoides* (95% CI)	Prevalence Hookworm (95% CI)	Arithmetic mean *epg* of Hookworm (95% CI)	Geometric mean *epg* of Hookworm (95% CI)	Prevalence of *T. trichiura* (95% CI)	Arithmetic mean *epg* of *T.s trichiura*	Geometric mean *epg* of *T. trichiura*
**Annual MDA**	Baseline (month 0)	792	46.3 (42.8, 49.9)	5754.9 (4683.1, 6826.7)	4267.8 (3619.1, 5032.9)	23.6 (20.7, 26.7)	125.0 (87.2, 162.7)	239.5 (201.0, 285.9)	36.5 (33.1, 40.0)	200.3 (166.1, 234.6)	286.8 (248.7, 330.7)
	Follow-up 1 (month 12)	729	49.5 (45.8, 53.2)	3016.5 (2340.5, 3692.5)	1692.8 (1412.2, 2029.1)	8.5 (6.5, 10.8)	37.7 (18.1, 57.4)	170.2 (121.4, 238.7)	8.6 (6.7, 10.9)	25.5 (14.8, 36.1)	133.3 (96.9, 183.3)
	Follow-up 2 (month 24)	473	57.9 (53.3, 62.4)	3629.3 (2874.4, 4384.2)	2081.3 (1718.2, 2524.1)	9.7 (7.2, 12.8)	16.6 (10.4, 22.8	110.3 (81.8, 148.6)	14.0 (11.0, 17.4)	28.1 (12.8, 43.5)	107.9 (85.5, 136.1)
	Follow-up 3 (month 36)	444	50.0 (45.2, 54.8)	1566.9 (1171.8, 1962.0)	917.2 (735.5, 1143.5)	8.8 (6.3, 11.8)	12.3 (5.8, 18.7)	72.6 (50.1, 105.1)	7.4 (5.2, 10.3)	5.9 (3.5, 8.4)	60.5 (45.2, 80.9)
	Follow-up 4 (month 72)	702	55.2 (51.51, 59.0)	1453.1 (1292.3, 1823.8)	729.3 (603.1, 910.3)	2.6 (1.4, 3.9)	5.2 (2.8, 7.3)	58.6 (45.9, 73.6)	1.9 (1, 3.1)	3.2 (1.6, 4.5)	43.6 (32.9, 68.7)
**Semiannual MDA**	Baseline (month 0)	698	37.7 (34.1, 41.4)	5302.6 (3966.2, 6639.0)	5140.5 (4274.8, 6181.6)	28.7 (25.3, 32.2)	214 (99.8, 328.3)	214.7 (176.5, 261.1)	38.4 (34.8, 42.1)	285.0 (150.4, 419.7)	236.9 (199.9, 280.7)
	Follow-up 1 (month 12)	624	34.1 (30.4, 38.0)	2043.4 (1499.1, 2587.8)	1366.2 (1056.4, 1766.8)	2.6 (1.4, 4.1)	7.4 (1.6, 13.3)	141.3 (71.4, 279.7)	8.5 (6.4, 11.0)	34.4 (-1.3, 70.0)	98.1 (68.3, 140.7)
	Follow-up 2 (month 24)	425	41.2 (36.5, 46.0)	700.5 (518.3, 882.7)	734.4 (601.1, 897.3)	13.2 (10.1, 16.8)	26.6 (14.4, 38.8)	105.0 (77.8, 141.6)	11.3 (8.4, 14.7)	38.7 (16.0, 61.4)	153.6 (108.4, 217.6)
	Follow-up 3 (month 36)	485	40.8 (36.4, 45.3)	1306.4 (912.3, 1700.4)	798.3 (627.8, 1015.2)	16.7 (13.5, 20.3)	41.8 (25.6, 58.0)	111.8 (84.8, 147.3)	9.1 (6.7, 12.0)	24.7 (6.3, 43.0)	80.6 (53.1, 122.1)
	Follow-up 4 (month 72)	467	66.8 (62.6, 71.3)	1055.6 (879.3, 1298.3)	648 (477.4, 983.5)	3.1 (1.8, 5.2)	11.5 (6.3, 16.4)	98.8 (74.2, 120.8)	6.8 (4.6, 9.3)	22.6 (4.6, 38.3)	68.2 (43.9, 97.2)

Arithmetic mean eggs per gram was calculated using data from all participants in the study, whereas geometric mean eggs per gram calculations was restricted to only those with at least one egg count.

Supplementary 2: Prevalence of STH and *S. mansoni* stratified by intensity levels (low and moderate/high intensity)

Treatment Zone	Year of survey	Intensity level	Prevalence of *Ascaris lumbricoides*	Prevalence of Hookworm	Prevalence of *Trichuris trichiura*	Prevalence of *Schistosoma mansoni*
Annual MDA	Baseline (month 0)					
		Low intensity	22.9	22.2	30.7	0.8
		Moderate/high intensity	23.5	1.4	5.8	0.0
	Follow-up 1 (month 12)					
		Low intensity	34.2	8.0	7.8	4.8
		Moderate/high intensity	15.4	0.5	0.8	0.5
	Follow-up 2 (month 24)					
		Low intensity	37.8	9.7	13.5	7.2
		Moderate/high intensity	20.1	0.0	0.5	0.8
	Follow-up 3 (month 36)					
		Low intensity	41.7	8.8	8.5	9.9
		Moderate/high intensity	8.3	0.0	0.6	0.0
	Follow-up 4 (month 72)					
		Low intensity	50.2	2.6	1.9	47.5
		Moderate/high intensity	5.2	0.0	0.0	0.7
Semiannual MDA	Baseline					

	(month 0)					
		Low intensity	17.5	27.1	32.2	0.1
		Moderate/high intensity	20.2	1.6	6.2	0.0
	Follow-up 1 (month 12)					
		Low intensity	24.4	2.6	8.2	0.2
		Moderate/high intensity	9.8	0.0	0.3	0.0
	Follow-up 2 (month 24)					
		Low intensity	36.9	13.2	10.5	0.5
		Moderate/high intensity	4.2	0.0	0.7	0.0
	Follow-up 3 (month 36)					
		Low intensity	33.6	16.7	8.5	3.3
		Moderate/high intensity	7.2	0.0	0.6	0.0
	Follow-up 4 (month 72)					
		Low intensity	62.3	3.1	6.8	46.1
		Moderate/high intensity	4.9	0.0	0.0	1.5

Intensity classes for egg counts

Ascaris infection: Low intensity; 1-4999 epg; Medium/high intensity >5000 epg
Hookworm infection: Low intensity; 1-1999 epg; Medium/high intensity >2000 epg
Trichuris infection: Low intensity; 1-999 epg; Medium/high intensity >1000 epg
Schistosomiasis mansoni infection: Low; 1-99 epg; Medium/high intensity >100 epg
